# The species composition, richness and diversity studies of fruit flies captured from the orchard of Nagpur mandarin

**DOI:** 10.1038/s41598-023-47299-y

**Published:** 2023-12-10

**Authors:** Sonal Nage, U. S. Kulkarni, D. B. Undirwade, N. M. Meshram, Swati Sant

**Affiliations:** 1 Department of Entomology, Dr. Panjabrao Deshmukh Krushi Vidhyapeeth, Akola, 444104 India; 2 ICAR-Central Citrus Research Institute (CCRI), Nagpur, 440033 India

**Keywords:** Entomology, Ecology, Evolution

## Abstract

The present experiment was conducted to study the diversity of fruit flies in Nagpur mandarin orchard from different agro-ecological zones of *Vidarbha* region of Maharashtra, India. The fruit flies samples were collected from 18 places falling under 9 agro-ecological zone using methyl eugenol traps. Out of the total collected catches of fruit flies from each zones, *Bactrocera dorsalis* was found to be the most prominent species followed by *Bactrocera zonata*. The *Z. cucurbitae* and *B. correcta* which occupied third and fourth rank in species composition, respectively. The species viz., *Zeugodacus duplicatus* and *Zeugodacus gavisus* recorded very less abundance at all locations. Shannons index of diversity was maximum in Morshi followed by Katol. Simpson index was more at Tiwasa and Achalpur locations which indicated minimum diversity in the population of fruit flies. Higher diversity of fruit flies were recorded in Morshi and Katol followed by Amravati.

## Introduction

Citrus is the native of South East Asia. It is one of the economically important fruits, occupies third rank next to mango and banana. Mandarin (*Citrus reticulata* Blanco) from Rutaceae family is considered to be one of the most important cultivated species among citrus which occupies nearly 40% of the total area under citrus cultivation in India. Maharashtra is the leading Mandarin producing State with 8.27 lakh tonnes of production^1^. Nagpur mandarin traditionally grown since past 300 years in central India comprising the *Vidarbha* region of Maharashtra and *Chhindwara* district of Madhya Pradesh^2^. Nagpur mandarin is finest variety and very popular in India as well as in world for its good quality fruits, attractive colour, size and the chemical properties of the juice i.e. its unique sweet–sour flavor, aroma and sugar acidity blends^3^. These characteristics are not found in any other orange across the world. Hence, it has been recently given the Geographical Indicators (GI) tag 385 in 2014 under the Geographical Indication of Goods, (Registration and Protection) Act 1999 on the basis of their unique qualities that can be attained only under specific soil and agro-climatic conditions of the *Vidarbha* region.

The Amravati and Nagpur districts of Maharashtra contribute about 80% of the total area under mandarin orchards in the state, sharing 48.88% and 31.45% respectively. With regard to the production of mandarin, Amravati district occupies 37.36% while Nagpur occupies 23.87% share in the *Vidarbha* citrus market. Further, *Vidarbha* is India’s only citrus-growing region with two fruiting seasons (*Ambia* and *Mrig*). Bangladesh is the biggest market for Nagpur mandarins. Nagpur and Amravati districts of Maharashtra together produce 7 lakh MT of Nagpur mandarin on 1.26 lakh ha area. Of this, Bangladesh alone imports 25% of the produce^4^.

In India, all the fruits of citrus are not drop at a time, they fall at various intervals. The loss occurs in a series of waves varying in the fruit sizes at different time interval. The shedding of citrus flowers and fruits occurs in three consecutive waves known as post-bloom drop or pre-harvest drop also known as June drop. Just after blossom, the tree lead to drop its small size fruits. A post-blossom drop results from unintentional over fruiting. Around 1–2 months after bloom, the second wave of fruit drops get started, due to the competition among fruit for energy (carbohydrates) for growth and development of young fruits. This drop is recorded particularly at summer or June during the course of fruit development due to high temperature and water scarcity. In the third wave, pre-mature and pre-harvest fruit drop includes the dropping of mature to harvestable fruits. The drop starts approximately from August, leading to fruit drop without the attached peduncle. In third wave many internal and external factors are responsible for the fruit drop. Now a day citrus cultivation is plagued with various problems due to limiting growing conditions, coupled with high incidence of insect pests and diseases and recently in the third wave, the fruit drop in citrus is occurred particularly due to fruit fly. This drop is of economic importance to the grower as almost fully grown fruits fall due to heavy fruit fly infestation, which affects the quality and quantity of fruit causing huge losses to the grower^5^.

Over 800 species of the tephritid fruit fly belong to the group Dacinae, which have been identified under the genus *Bactrocera* and *Dacus*. About 60 of these are known to exist in India, primarily infesting fruits and vegetables and leading to yield losses^6^. Based on location, variety, and season, tephritid fruit flies directly cause damage to important export crops including mango, avocado and cucurbits, resulting 40% to 80% losses^7^. Fruit flies induce fruit ripening, destroy the fruit’s pulp and results premature fruit drop. During 2000 and 2001 citrus fruits were attacked by fruit flies varied between (5 and 70) % and (5 and 60) % respectively, in Nigeria^8^. According to^9^ the fruit flies are directly damaging the fruit crops which may lead to losses of 40–80% or even more, the management of fruit flies is challenging because their life-stages occur at different sites and are unexposed, e.g. eggs and larvae in the host, pupae in soil and adults are active flier. They infests large number of host plants with many generations in a year, adults have high mobility and long life span as they can live for more than 3 months. Besides, a single female can lay more than 1000 eggs. Also the use of formerly effective broad-spectrum and systemic insecticides is not recommended against fruit flies because of consumers’ reactions.

The present study was undertaken with an aim to know the diversity, species richness and evenness of fruit flies in major citrus growing area of *Vidarbha*. The data generated in species composition of fruit flies will help to understand the region wise variability in fruit flies species, obtain the knowledge on its habitat and behavior prior to applying any pest management strategy.

## Materials and methods

The present studies was undertaken in Nagpur mandarin orchards during *Ambia bahar* from June 2022 to November 2022. Different agro ecological zones of *Vidarbha* were selected i.e. Akola, Amravati, Achalpur, Morshi, Chandur bazar, Warud, Katol, Arvi, Tiwasa region of Maharashtra. The orchards were randomly chosen from the respective places and two places from each zone were selected for the collection of fruit flies. Total 18 places were covered from nine agro-ecological zones. Fruit fly traps were hanged at each selected site at 3 m above the ground surface. The methyl eugenol lure was used to attract the flies and the lure was refreshed at monthly interval. The flies trapped in the traps were collected carefully from each location, brought to laboratory and counted. The collected fruit flies sample were critically examined under a binocular microscope and sorted out in to different species on the basis of morphological characters by applying ‘Tephritid Key’ (Given by: Dr. C. A. Viraktamath and Dr. K. J. David, UAS, GKVK, Bangalore) under different code for taxonomic identification. Accordingly, the data on fruit flies species at each location was documented. For the further confirmation the fruit flies species sample was send to ICAR-National Bureau of Agricultural Insect Resources (NBAIR), Bangalore.

The data was used for further analysis of diversity and species richness. Species richness (a count of different species in a given area) and diversity was assessed through Shannon’s diversity index^10^ using following formulas.

$$\text{Shannon Index }(\text{H})=-\sum_{i=1}^{s}{p}_{i}\,In\,{p}_{i}$$

where; H = Shannon’s diversity index; Pi = Fraction of total population belonging to species *i*, S = Total number of species (species richness).

The Simpson's index is the probability that any two individuals randomly selected from finite size community will belong to the same species^11^.

$$\text{Simpson's index } (D)=\sum \left(\frac{{n}_{i}\left[{n}_{i}-1\right]}{N\left[N-1\right]}\right)$$

where: n_i_—number of individuals in the *i*-th species; and N—total number of individuals in the community.

Values of D range from 0 to 1; increase in D value shows a decline in diversity. Therefore, reciprocal form of Simpson’s index (1/D) is usually adopted and in the present study also the same was used as the index of diversity^12^.

To know the measure of how similar the abundance of different species, species evenness was calculated to estimate the equitability component of diversity^10,13^.

$$\text{Species Evenness } J=\frac{H}{{\mathrm{log}}_{e}S}$$

where, H is the Shannon–Wiener biodiversity index, S is the number of species in the community.

## Results and discussion

**Figure 1:**
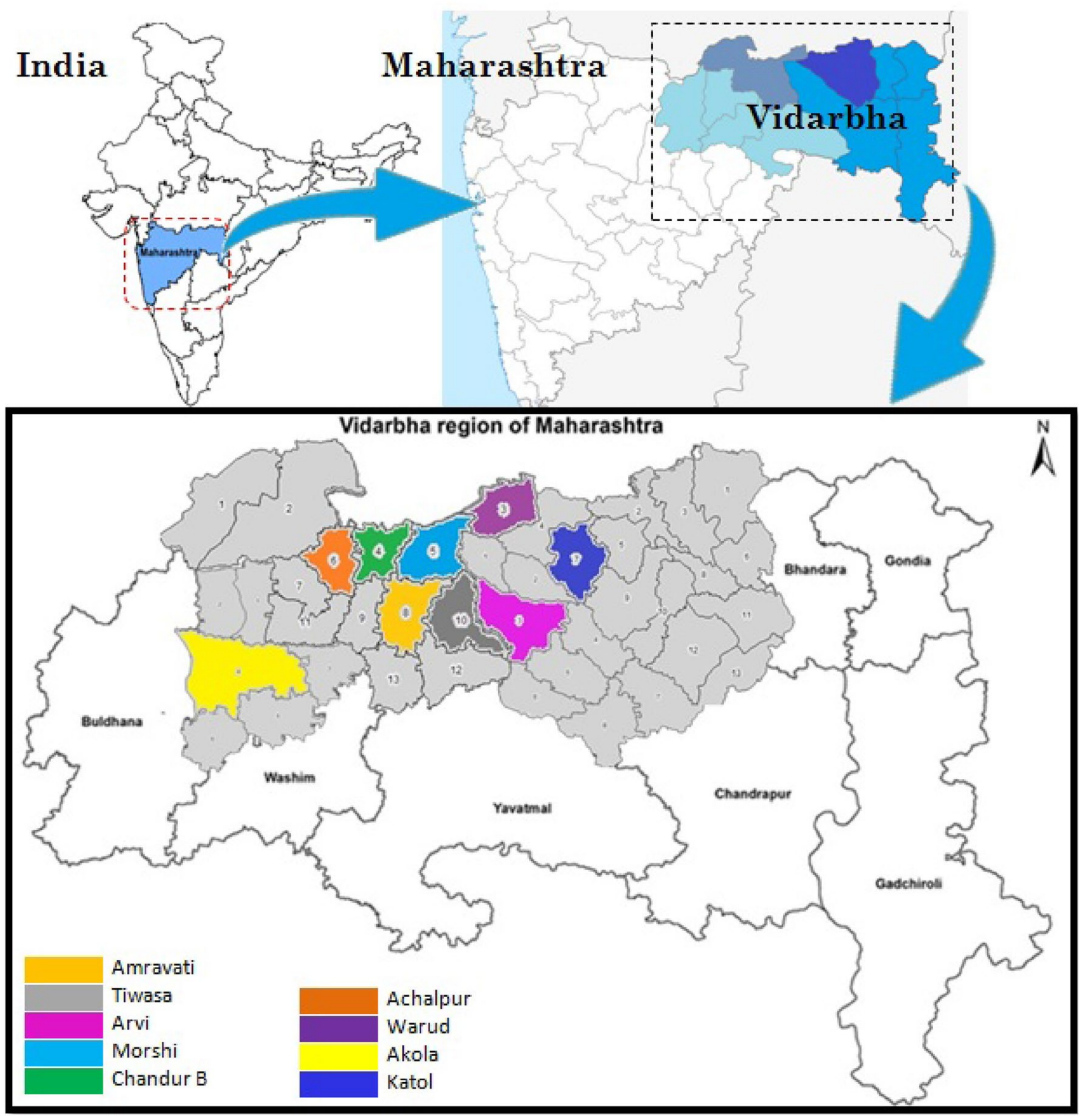
Sampling sites at different agro-ecological zone of Vidarbha region of Maharashtra.

**Figure 2:**
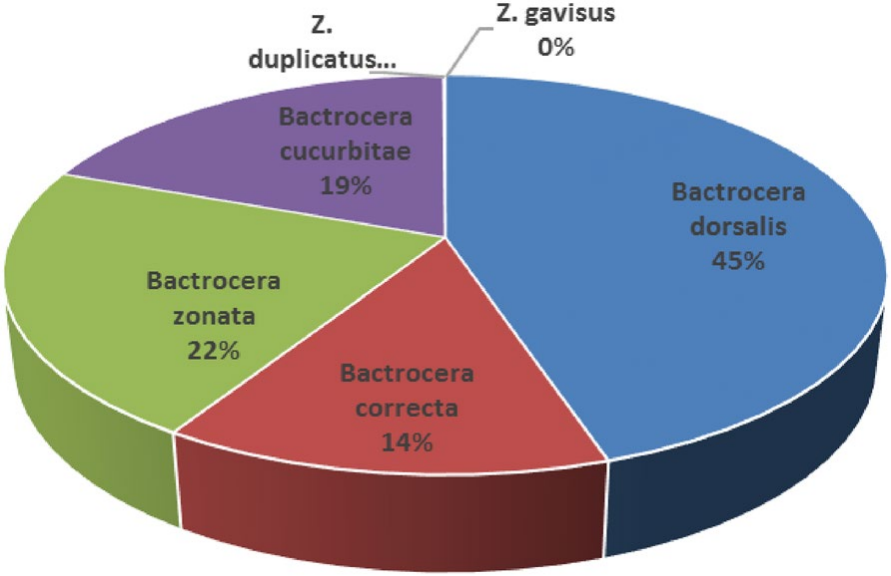
Relative abundance (%) of Fruit fly species found in the different agro-ecological zone of Vidarbha region of Maharashtra.

During the experiment, seven fruit fly species from Tephritidae families were collected through methyl eugenol trap in Nagpur mandarin orchard from different agro ecological zones of Akola, Amravati, Achalpur, Morshi, Chandur bazar, Warud, Katol, Arvi and Tiwasa region of *Vidarbha* (Maharashtra) are presented in Fig. 1. As a result of present studies total 19,252 fruit flies were collected out of these the most predominant species were *B. dorsalis* (45%), followed by *B. zonata* (22%), *Z. cucurbitae* (19%) and *B. correcta* (14%) in Fig. 2. The species *Zeugodacus duplicatus* and *Zeugodacus gavisus* were very less abundant in all locations and reported as Non-pest. All the identified species were previously known to be attracted to methyl eugenol traps except the species *Z. cucurbitae* and *Z. gavisus,* which were previously known to be attracted to cue lure*.* However, according to Refs.^14,15^, the abundance and occurrence of fruit fly species in mango and citrus ecosystems may vary depending on biotic and abiotic factors, type of food, and geography of the location.

**Figure 3:**
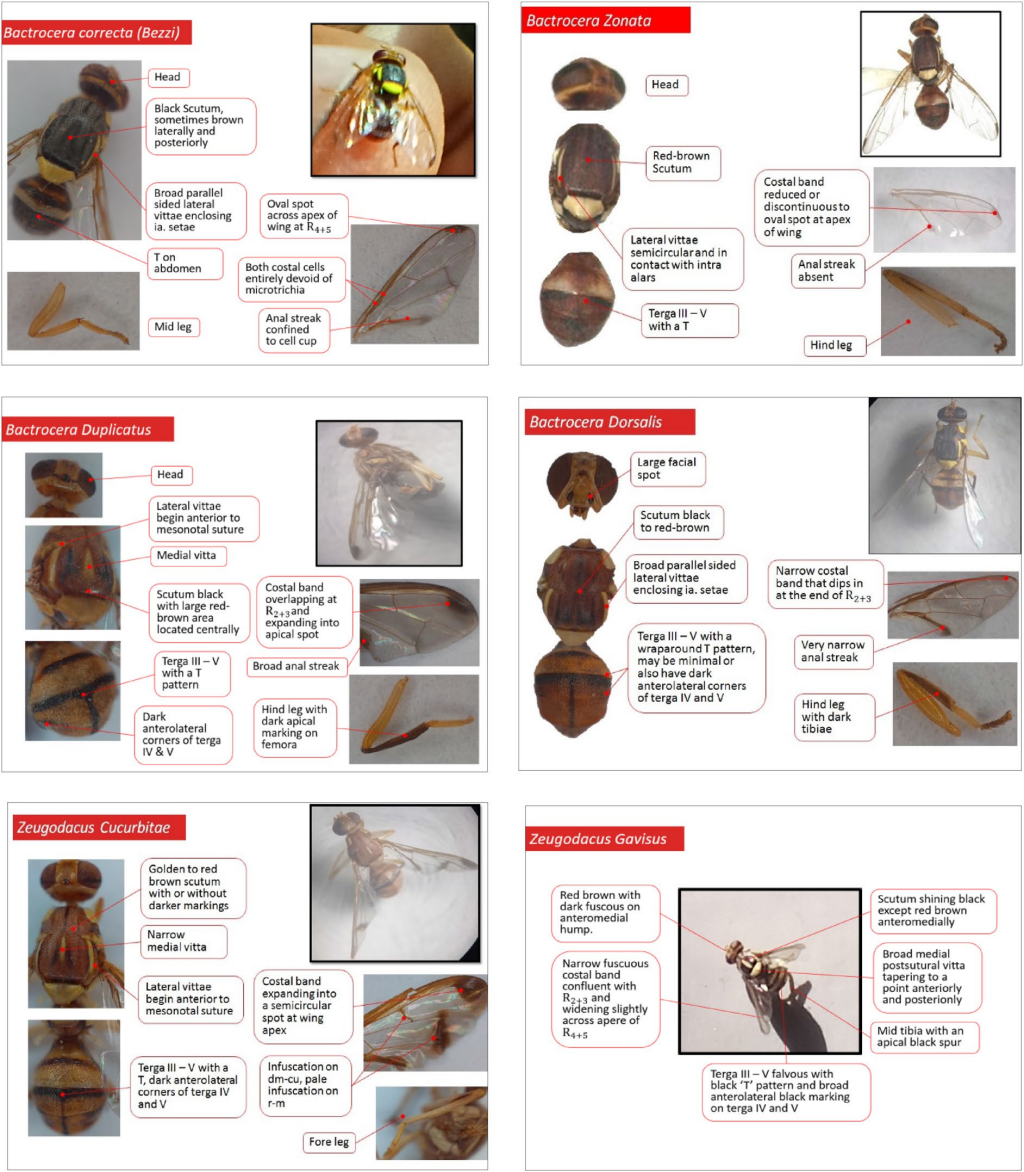
Morphological description of Fruit fly species identified from the different agro-ecological zone of *Vidarbha* region of Maharashtra.

The fruit flies, viz. *Zeugodacus gavisus* recorded only from Arvi taluka of Wardha district and *Zeugodacus duplicatus* were recorded from Arvi and Tiwasa taluka of Wardha and Amravati district, respectively. In general at all the locations, a comparably higher population of *B. dorsalis* was recorded which were followed by *B. zonata*. The Arvi location shown the highest diversity with six species which is followed by Tiwasa with five species. As compare to Arvi and Tiwasa locations, remaining all the locations had a lower diversity with only four species. The morphological description of six identified species has been described in Fig. 3. The species complex in descending order were *Bactrocera dorsalis* (8665 flies) > *Bactrocera zonata* (4210 flies) > *Zeugodacus cucurbitae* (3700 flies) > *Bactrocera correcta* (2660 flies) > *Zeugodacus duplicatus* (13 flies) > *Zeugodacus gavisus* (4 flies) are represented in Table 1.

**Table 1:** Abundance of fruit flies collected from the different agro-climatic zones of *Vidarbha* region of Maharashtra.

Locations	Species abundance of fruit flies (ni)						
	*Bactrocera dorsalis*	*Bactrocera correcta*	*Bactrocera zonata*	*Zeugodacus cucurbitae*	*Z. duplicatus*	*Z. gavisus*	Total (Ni)
Akola	830	320	280	400	0	0	1830
Amravati	410	270	250	120	0	0	1050
Tiwasa	1030	250	310	200	5	0	1795
Achalpur	1270	220	480	270	0	0	2240
Chandur B	1190	280	720	370	0	0	2560
Morshi	1120	410	580	700	0	0	2810
Warud	1165	350	660	450	0	0	2625
Katol	450	230	310	160	0	0	1150
Arvi	1200	330	620	1030	8	4	3192
Total	8665	2660	4210	3700	13	4	19,252

Fruit fly diversity indices and richness were calculated for each agro-ecological zone. Morshi location was the highest Shannon index i.e. (H = 1.319), followed by Katol (H = 1.317). Shannon index, presumes that each species is present in the sample and that it was drawn at random. Both Tiwasa and Achalpur locations were the higher Simpson indexes (D = 0.391), indicating that fruit fly diversity was low. Fruit fly diversity was highest in the areas of Morshi and Katol (D = 0.285), then Amravati (D = 0.288) as given in Table 2. The Simpson index is a measure of dominance, because it provides higher weight to prevalent or dominant species. Biodiversity (diversity index, species richness and evenness) of fruit flies in Morshi and Katol, followed by Amravati were more than the remaining sites. Even though there were no mandarins in the orchard, fruit flies were still present in greater numbers, which suggested that they might be attracted to the late-season fruits found in commercial orchards or other host crops present in the area, such as vegetables.

**Table 2:** Diversity indices of species of fruit flies collected from the different agro-ecological zones of *Vidarbha* region of Maharashtra.

Sr. no.	Locations	Shannons index (H)	Species richness	Simsons index (D)	Species evenness (J)	Simpson reciprocal index (1/D)
1	Akola	1.283	4	0.307	0.716	3.256
2	Amravati	1.306	4	0.288	0.729	3.476
3	Tiwasa	1.157	5	0.391	0.646	2.560
4	Achalpur	1.135	4	0.391	0.633	2.556
5	Chandur B	1.234	4	0.328	0.689	3.051
6	Morshi	1.319	4	0.285	0.736	3.514
7	Warud	1.279	4	0.307	0.714	3.256
8	Katol	1.317	4	0.285	0.735	3.515
9	Arvi	1.309	6	0.294	0.731	3.405

All the identified species were belonged to the genus *Bactrocera* and *Zeugodacus* under family Tephritidae. These results are supported by studies by Madhura and Viraktamath^16^ who identified five species of fruit flies that were attracted to methyl eugenol: *B. dorsalis, B. correcta, B. verbascifoliae, B. affinis, and B. zonata*. In the Konkan region, Morde^17^ hanged methyl eugenol traps in mango crop and recorded the fruit fly species *B. caryeae, B. dorsalis, and B. zonata* caught in trap*.* The *B. correcta, B. dorsalis, and B. zonata* incidences were also recorded by Kawashita et al.^18^ from Sri Lanka. In the coastal zone of guava ecosystems, Satarkar et al.^19^ found that methyl eugenol attracted four species of fruit flies in guava orchard, including *B. caryeae, B. zonata, B. affinis, and B. correcta.* The present investigation was also ratify by the report of Ukey et al.^20^, *B. dorsalis* was the most abundant species in guava ecosystem reported from the Ahmednagar area of Maharashtra, followed by *B. zonata and B. correcta*. Similar to this, Nagaraj et al.^21^, B. dorsalis was the most predominant species reported in mango ecosystem of GKVK campus, comprising 49.41 percent, followed by *B. correcta*, which occupied a 34.22 percent abundance. The present findings are similar to those of Stonehouse et al.^22^ and Deepa et al.^23^, Ravikumar^24^ in guava and mango orchard of Dharwad region and Galande and Ukey^25^ in guava orchard of Pune region, reported that *B. dorsalis*, *B. correcta*, and *B. zonata* were caught in methyl eugenol traps.

The present study was undertaken in order to investigate the species diversity and abundance of fruit flies in the different agro-ecological zones of the *Vidarbha* region in Maharashtra. These results are confirmed by the reports of Win et al.^26^, who recognized eleven species of fruit flies from Mango, Guava and Jujube in Myanmar and observed that *B. correcta* (29.3%) and *B. dorsalis* (28.6%) were the two most prevalent species among all the species that emerged as adults from various fruit samples. The four fruit fly species *B. dorsalis, B. zonata, B. correcta, and B. diversa* that were captured in to methyl eugenol traps from mango orchard of Western plain zone of Uttar Pradesh were identified by Kumar et al.^27^. They reported that only *B. dorsalis* was predominate on mango orchards in both the Saharanpur district and Meerut district (U.P.) locations. The outcomes of the present study are also in line with those of Math et al.^28^, who investigated the diversity of fruit flies in Bagalkot region and reported that *B. correcta, B. dorsalis* and *B. zonata* were the predominant species and *B. duplicata* was quite rare in the guava ecosystem. According to Kapoor^29^, the most significantly the fruit fly pest complex in India, consists of B*. correcta, B. dorsalis,* and *B. zonata* species from mango, guava and cucurbits fields. Vanitha et al.^12^ revealed that methyl eugenol attracted *B. dorsalis* (71.66%), *B. correcta* (23.70%) and *B. zonata* (4.50%) from mango orchard at different agro-climatic zones of Karnataka. Irsad and Haseeb^30^ identified *B. zonata, B. dorsalis *and* B. correcta* and reported that *B. zonata* exhibited a significantly higher population in the guava-growing regions in western Uttar Pradesh.

From Nagpur Mandarin orchard in *Vidarbha* region of Maharashtra, total six fruit flies species were identified. Out of total collected catches of fruit flies, *B. dorsalis* was found to be most prominent species with 45% abundance followed by *B. zonata* with 22%. The *Z. cucurbitae* and *B. correcta* constituted 19% and 14% abundance, respectively. The *B. zonata, Z. cucurbitae and B. correcta* occupied second, third and fourth rank in composition, respectively. The species *Z. duplicatus* and *Z. gavisus* were very less abundant in all locations. The Morshi and Katol regions had the greatest diversity, after that the next one was Amravati.

## Data Availability

The data generated during the study are not publicly available because it is the results of our own research that has not yet been published anywhere. That data generated from raw data which is available from the corresponding author on reasonable request.
